# Genome Sequence of *Arthrobacter globiformis* Bacteriophage TrixiePhattel

**DOI:** 10.17912/micropub.biology.001993

**Published:** 2026-07-02

**Authors:** Christina I. Hernandez, Tyler J. Potter, Luke T. Mumaw

**Affiliations:** 1 Life Science, Mesa Community College, Mesa, AZ, United States

## Abstract

We report the morphology and genome of a novel bacteriophage. TrixiePhattel, isolated from silty soil collected at the bank of a creek in Potato Patch AZ, USA, is capable of infecting *Arthrobacter globiformis* ( *A. globiformi* s), and possesses a siphovirus morphology. Due to gene content, TrixiePhattel is assigned to the actinobacter phage cluster AU6. The absence of identifiable genes involved in lysogeny and failure to raise lysogens suggests TrixiePhattel is a virulent phage.

**Figure 1. Negative-stain transmission electron micrograph of TrixiePhattel:** Negative-stain (uranyl acetate, 1%) transmission electron microscopy of TrixiePhattel revealed a siphovirus morphology with a prolate capsid characteristic of AU6 cluster phages. Scale bar is 200 nm (x60.0k Zoom-1 HIR-1 100.0kV).

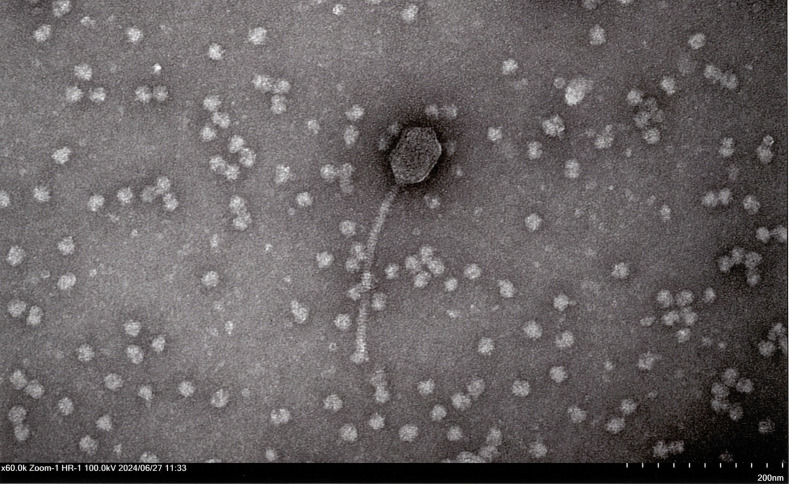



## Description

Bacteriophages are viruses that infect bacteria and represent a highly abundant and diverse component of the biological world (Hatfull, 2022). Due to their ability to infect and kill specific bacterial hosts they are increasingly being developed as therapeutics for antibiotic-resistant bacterial infections (Sawa et al., 2024). Given that phages have narrow host ranges, the isolation and characterization of novel bacteriophages is an important aspect of this effort. To this end we report the discovery of a new bacteriophage, TrixiePhattel, collected from silty soil at the bank of a creek in Potato Patch AZ, USA (34.432769 N, 112.415035 W) which can infect the bacterium *Arthrobacter globiformis* ( *A. globiformis* ). To date, TrixiePhattel is one of less than ten of phages isolated in the state of Arizona that has been sequenced (Russell & Hatfull, 2016), offering insights into phages from arid environments.

TrixiePhattel was isolated using standard procedures (Zorawik et al., 2024). Briefly, silty soil collected at the bank of a creek in Potato Patch AZ, USA (GPS Coordinates 34.432769 N, 112.415035 W) was suspended in PYCa (peptone, yeast extract, calcium) liquid medium, the suspension filtered (0.02 um pore size), and the filtrate inoculated with *A. globiformis* and incubated at 30˚C for 20 hours with shaking. The resulting culture was spun, the supernatant filtered, and the filtrate plated in PYCa top agar supplemented with *A. globiformis* . TrixiePhattel formed small clear plaques and was purified with two rounds of plating. Negative stain (1% uranyl acetate) transmission electron microscopy revealed a siphovirus morphology measured using ImageJ version 1.54 (Schneider et al., 2012) with a prolate head of 84x54nm and a tail of 216x11nm (n=1; Figure 1).

DNA was isolated from a filtered lysate of TrixiePhattel and sequencing was performed on an Illumina NextSeq 1000 (XLEAP-P1 kit), with libraries prepped using the NEB Ultra II FS kit. A total of 3.9M 150-base raw reads were trimmed with Cutadapt v. 4.7 (using the option: –nextseq-trim 30) and filtered with Skewer v. 0.2.2 (using the options: -q 20 -Q 30 -n -l 50) prior to assembly using Newbler v. 29 (https://bio.tools/newbler) (Russell, 2017) to generate a single genome with 9,566-fold coverage. Consed v. 29 was then used to check for correctness of joined sequences (Gordon et al., 1998). The genome is 55,228 base pairs long with a GC content of 49.9% and 9 bp 3’ single-stranded overhang of 5’ CGCCGCCCT.

Glimmer v. 3.02 (Delcher et al., 1999), and Genemark v. 2.5p (Besemer & Borodovsky, 2005) was used to auto-annotate the bacteriophage genome in DNA Master (Pope & Jacobs-Sera, 2018). The auto-annotation was refined using Genemark, Phamerator (using Actino_draft database v578) (Cresawn et al., 2011), Starterator (phages.wustl.edu/starterator/), and BLAST (Sayers et al., 2025)using the Actinobacteriophage and NCBI non-redundant databases. Gene function was assigned in PECAAN (https://discover.kbrinsgd.org) using HHPred searches against the PDB_mmCIF70, Pfam- v.36, NCBI Conserved Domains databases (Söding et al., 2005) and transmembrane domain containing proteins predicted with DeepTMHMM v. 1.0.42 (Hallgren et al., 2022). Default parameters were used for all software.

We were able to assign putative functions to 31 of the 93 predicted genes, and three of the unassigned genes are orpham genes currently unique to TrixiePhattel. Based on gene content similarity of at least 35% to phages in the Actinobacteriophage database, phagesdb, TrixiePhattel was assigned to cluster AU6 (Pope et al., 2017; Russell and Hatfull, 2016) with a closest percent identity of 84.98% with the AU6 cluster phage KevinMinion. As with other AU6 phages, all genes are rightward transcribed unidirectionally. In contrast to the synteny of most siphoviridae phages where both endolysin and holin genes are found after the minor tail protein genes, in TrixiePhattel and other AU6 cluster phages, the predicted gene for endolysin comes before the genes for virion structure and function. No known AU6 cluster phages have identifiable tRNA’s, integrases, or repressor functions, the latter suggesting that AU6 phages are unlikely to establish lysogeny, which is consistent with unsuccessful efforts to raise lysogens (Wise & Sivanathan, 2025).

**Nucleotide sequence accession numbers**

TrixiePhattel at GenBank with Accession No. PV876940 and Sequence Read Archive (SRA) No. SRX31241833. 
